# How to 19F MRI: applications, technique, and getting started

**DOI:** 10.1259/bjro.20230019

**Published:** 2023-09-29

**Authors:** Olga Maxouri, Zuhir Bodalal, Mariah Daal, Sajjad Rostami, Ivonne Rodriguez, Leila Akkari, Mangala Srinivas, René Bernards, Regina Beets-Tan

**Affiliations:** 1 Department of Radiology, The Netherlands Cancer Institute, Amsterdam, The Netherlands; 2 GROW School for Oncology and Developmental Biology, Maastricht University, Maastricht, The Netherlands; 3 Department of Cell Biology and Immunology, Wageningen University & Research, Wageningen, The Netherlands; 4 Cenya Imaging BV, Amsterdam, The Netherlands; 5 Division of Tumor Biology and Immunology, Oncode Institute, The Netherlands Cancer Institute, Amsterdam, The Netherlands; 6 Division of Molecular Carcinogenesis, Oncode Institute, The Netherlands Cancer Institute, Amsterdam, The Netherlands

## Abstract

Magnetic resonance imaging (MRI) plays a significant role in the routine imaging workflow, providing both anatomical and functional information. 19F MRI is an evolving imaging modality where instead of 1H, 19F nuclei are excited. As the signal from endogenous 19F in the body is negligible, exogenous 19F signals obtained by 19F radiofrequency coils are exceptionally specific. Highly fluorinated agents targeting particular biological processes (*i.e.,* the presence of immune cells) have been visualised using 19F MRI, highlighting its potential for non-invasive and longitudinal molecular imaging. This article aims to provide both a broad overview of the various applications of 19F MRI, with cancer imaging as a focus, as well as a practical guide to 19F imaging. We will discuss the essential elements of a 19F system and address common pitfalls during acquisition. Last but not least, we will highlight future perspectives that will enhance the role of this modality. While not an exhaustive exploration of all 19F literature, we endeavour to encapsulate the broad themes of the field and introduce the world of 19F molecular imaging to newcomers. 19F MRI bridges several domains, imaging, physics, chemistry, and biology, necessitating multidisciplinary teams to be able to harness this technology effectively. As further technical developments allow for greater sensitivity, we envision that 19F MRI can help unlock insight into biological processes non-invasively and longitudinally.

## Review outline

19F MRI is a versatile tool in biomedicine with the ability to probe biological processes non-invasively using fluorinated agents. This review intended to outline the various aspects of 19F MR imaging with a beginner imaging researcher (non-MR physicist) in mind. While we do not engage in an exhaustive review of all 19F literature, we aim to showcase work that might be relevant to imaging/research teams starting out and will refer readers regularly to dedicated reviews/articles that would delve deeper into given topics.

This article can be generally divided into three sections. Section I provides background on 19F MRI in medical imaging and highlights different applications. Examples are provided in different disease models, with a focus on cancer imaging. Section II aims to point the reader towards practical considerations for 19F MRI studies. It discusses the main components of 19F MRI together with common pitfalls a beginner may encounter. Section III summarises these developments within this rapidly evolving research field and highlights their potential impact on future research.

## Section I: Background and applications of 19f MRI

### Background

Technological advancements in molecular imaging have greatly shaped how clinicians approach patient care and research. Biological targets and metabolic processes can be quantitatively and non-invasively visualised, offering a biological context to the anatomical and functional imaging already available to radiologists. Molecular imaging carries great promise for the emerging field of precision medicine, which tailors treatment to the patient’s unique biology. For instance, molecular imaging in atherosclerosis, Alzheimer’s disease, and cancer may be leveraged to identify early-stage pathological changes, improving detection and aiding in treatment response evaluation.^1–3^ Early detection is not only key in reducing both morbidity and mortality in patients, it could also enable the enrolment of pre-symptomatic patients in clinical trials improving drug discovery efforts.^4,5^

Traditionally, single photon emission computerised tomography (SPECT) and positron emission tomography (PET) have been the modalities of choice for molecular imaging due to their exceptional sensitivity, which allows the detection of radiotracers at concentrations in the nanomolar (10^−9^) to the picomolar range (10^−12^). 18F-FDG is used routinely in the clinic to visualise altered metabolism in cancer and neurodegenerative dementias.^6,7^ However, SPECT and PET come with their own set of drawbacks. Clinical SPECT suffers from poor spatial resolution and has a lower sensitivity than PET (two orders of magnitude). Moreover, PET is more expensive and less accessible as tracers require a sophisticated radiopharmaceutical facility to conjugate the radioisotope to the targeting molecule/antibody.^8,9^ PET tracers also exhibit fast radioactive decay, limiting the window of opportunity to scan and potentially exposing the targeted cells to damaging radiation.

Another modality in molecular imaging is magnetic resonance imaging (MRI), characterised by unlimited tissue penetration, excellent soft-tissue contrast, and high spatial resolution without exposing the patient to ionising radiation. Conventional 1H MRI is widely used in clinical settings to generate both anatomical and functional images. Different 1H MRI techniques have been associated with biological processes.^10–12^ Relevant targets of molecular 1H MRI include deoxyhaemoglobin, metabolites, proteins, and lipids. For example, blood-oxygen-level-dependent (BOLD) MRI reveals oxygen availability in different regions, which can provide insights into tumour hypoxia, chronic kidney disease, and myocardial ischaemia.^13–15^ Another technique, multivoxel magnetic resonance spectroscopy, spatially resolves metabolic data important for clinical decision-making in stroke, neurodegenerative diseases and brain cancer.^10,16^

Although 1H MRI can probe biomolecular processes, it lacks specificity as hydrogen nuclei are ubiquitously present in the body, irrespective of physiology. The use of contrast agents also introduces a unique set of challenges. To visualise the impact of the contrast agent, radiologists require ‘before and after’ images of which agent concentration is difficult to quantify.^17^ Concerns have also risen regarding contrast toxicity, which remains to be fully elucidated.^18,19^ For example, superparamagnetic iron oxides (SPIOs) create hypointense signals on *T*_2_-weighted images, which may be hard to distinguish from blood. Moreover, SPIOs administered for sentinel lymph node detection in breast cancer may impair MRI examinations due to void artefacts remaining in the tissue for many years.^20^

X-nuclei MRI complements 1H MRI as it examines other non-proton nuclei with a magnetic moment to visualise biological processes. A specifically tuned radiofrequency (RF) coil allows target nuclei to be excited and visualised in an image. Nuclei of interest may be endogenous (*e.g.,* 23Na, 35 Cl and 39K) or exogenous (*e.g.,* 19F) to the human body. Endogenous nuclei reveal metabolic and physiological information in health and disease.^21,22^ In addition, exogenous nuclei can be incorporated into contrast agents or other molecules allowing the labelling of drugs and cells or the visualisation of metabolic processes. The clinical use of X-nuclei lags behind 1H MRI, mainly due to sensitivity limitations. It is further limited by its high cost, requiring trained staff and specialised equipment, and lack of diagnostic value. As MRI magnetic field strength and hardware setups have improved, so has the SNR of X-nuclei, paving the way for explorative studies.^23,24^

The 19F nucleus is a promising target for imaging (Table 1). 19F MRI exhibits high specificity *in vivo* since the body contains trace amounts of fluorine, predominantly immobilised in the bone matrix and tooth enamel, rendering it undetectable. As such, 19F MRI can be performed in a background-free setting with a high contrast-to-noise ratio (CNR). In terms of quantitative MRI, this is a major advantage over 1H MRI contrast agents such as SPIOs because the MR signal intensity directly reflects the concentration of 19F nuclei. In addition, 19F is more sensitive than 1H to changes in the local chemical environment and displays a wide chemical shift range. This characteristic allows for the design of theranostic MRI probes and probes reactive to changes in the local environment (*i.e.,* stimuli-responsive).^25^

**Table 1.** A general overview of the advantages and disadvantages of 19F MRI

Advantages	Disadvantages
Absence of ionising radiation	Low SNR due to intrinsically low sensitivity of MRI (19F concentration in the millimolar range)
Non-invasive, longitudinal imaging possible	Long scan times, caused by low 19F concentration in tissues and long T1 of some 19F agents
High MR sensitivity: 83% of 1H	Requires hardware modifications and specialised coils
Quantitative in nature	High uptake of 19F probes by the reticuloendothelial system
Absolute specificity: background-free imaging	Some probes have a long biological half-life
Multispectral imaging possible	
19F MRI agents are responsive to their environment: possibility to probe biological processes	

#### The challenge of sensitivity

The sensitivity of MRI is lower than other molecular imaging techniques and to successfully acquire a 19F MR image, the 19F concentration must be in the millimolar range (10^−3^).^26,27^ With 1H MRI, this is less of an issue due to the high water content in the body, which is approximately 50–60% for the average adult (~55.6–66.7 M of hydrogen). The low sensitivity poses a challenge to experiments where the goal is to visualise targets *in vivo* which are present at low concentrations (*e.g.,* enzymes) or specific cell populations. Different strategies have been implemented to improve the sensitivity of 19F MRI, such as increasing the main magnetic field strength, the 19F probe payload (*e.g.,* perfluorocarbons), altering the relaxation properties of 19F through intramolecular interactions with lanthanides, and adjusting both acquisition and data processing procedures.

### Applications of 19F MRI

**Figure 1.**
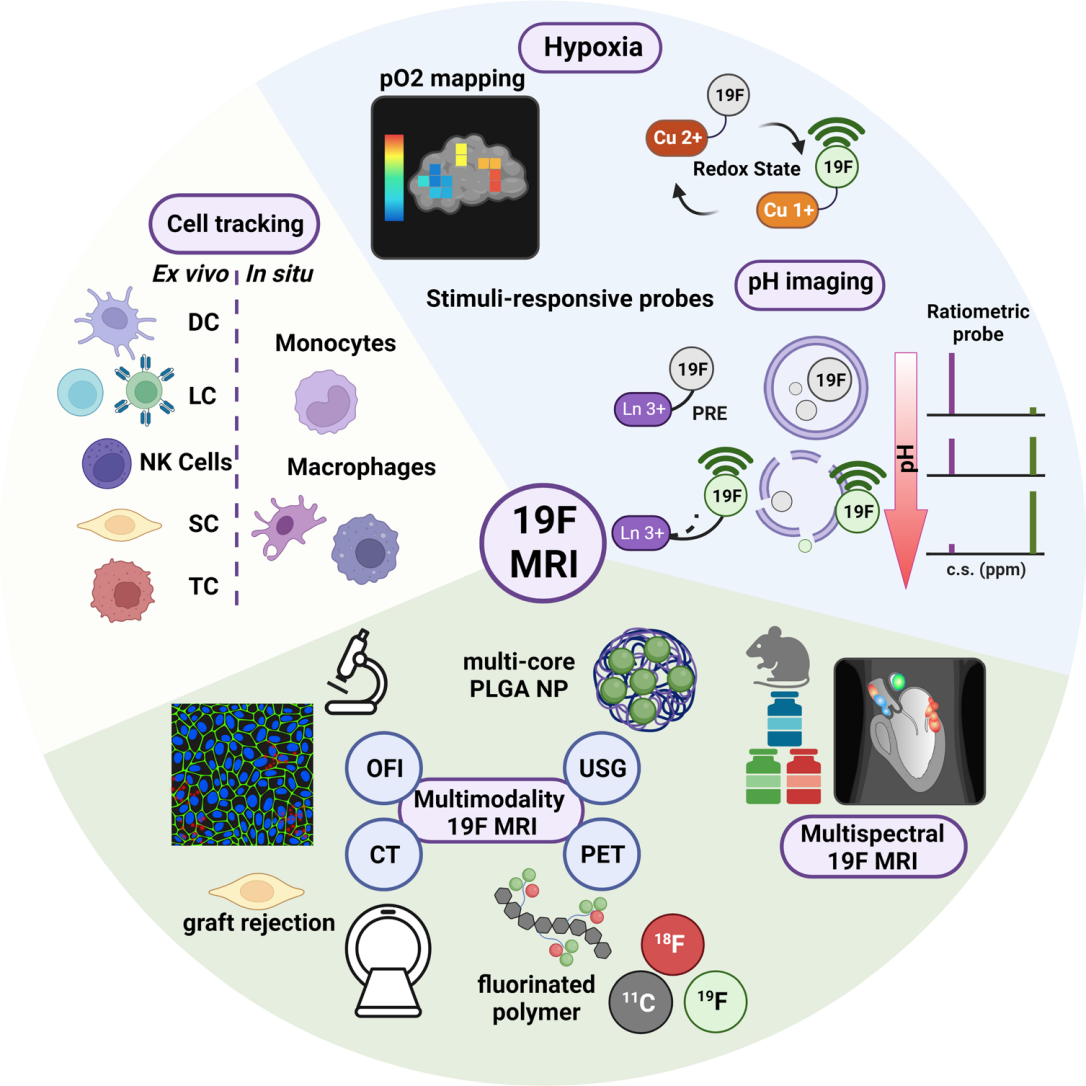
Highlights of 19F MRI for biomedical applications. Cell tracking and quantification play a prominent role in 19F MRI research due to the quantitative nature and specificity of 19F MRI. PFC formulations have been used to label cells *ex vivo* and *in situ* in order to probe the fate of transplanted cells or to image inflammation. Molecular 19F MRI non-invasively probes physiological parameters such as hypoxia, pH, and redox state. Stimuli-responsive OFF/ON nanoprobes attenuate the MR signal of neighbouring 19F atoms through their influence on the local environment. Silencing of 19F reporters occurs via T2 shortening, which can be achieved by immobilising 19F spins or via PRE. Conformational changes or probe disassembly is induced in the presence of the desired stimuli, thereby restoring T2 along with the 19F MR signal. The fate of 19F agents can be further influenced by functional groups and the structure of a nanocarrier system (*e.g.,* multicore PLGA NP). In addition, multimodality and multispectral MRI add another dimension of information to 19F MRI studies. Fluorescent moieties incorporated in 19F formulations allow subsequent model characterisation via *ex vivo* cytometric analyses such as flow cytometry and fluorescence microscopy. Radioisotopes combined with fluorinated polymers enable sensitive PET imaging alongside 19F MRI. Different cell populations or targets can be imaged within a single subject with multispectral MRI using 19F agents with discriminating NMR signals. c.s: chemical shift, CT: computed tomography; DC: Dendritic cell; LC: lymphocyte; NK cell: natural killer cell; OFI: optical fluorescence imaging; PET: positron emission tomography; PFC: perfluorocarbon; PLGA NP: poly(lactic-co-glycolic acid) nanoparticles; pO2: partial oxygen tension; PRE: paramagnetic relaxation enhancement; SC: stem cell; TC: tumour cell; USG: ultrasound sonography

In 1977, Holland et al. generated the first 19F MR images shortly after the first anatomical 1H MR images.^28^ Since then, 1H MRI has developed into an integral medical imaging tool. Although the clinical translation of 19F MRI did not share the same trajectory, various applications have been explored pre-clinically, including imaging of (immune) cells, angiogenesis, tumour acidosis, and hypoxia.^29–32^ Figure 1 highlights the applications of 19F MRI which will be discussed in the following subsections, with the exception of multispectral 19F MRI (SectionIII).

#### Cell tracking

Cell-based therapies are a burgeoning approach to fighting disease and restoring organ function. Stem cells may be deployed for the regenerative treatment of cartilage, the heart and the pancreas and to combat inflammation.^33^ Cellular therapies are also promising options for cancer treatment, with possible durable responses in patients with advanced and treatment-refractory tumours.^34–36^ Several factors contribute to the success of cellular therapies, including correct homing to the target lesion or lymphoid organ, cell survival, and proliferation.^37,38^ Non-invasive tracking of these cells via imaging would therefore be beneficial for translating and applying cellular therapies.

19F nanoprobes have been studied extensively in the context of cell labelling and tracking.^29,39–42^ Cell tracking is achieved through the *ex vivo* or *in vivo* labelling of cells with perfluorocarbon (PFC) nanosystems, allowing for the collection of spatio-temporal and quantitative information.^43–46^ Since 19F MRI only visualises exogenous 19F atoms, highly specific “heatmaps” can be generated.

*Ex vivo* cell labelling with PFC nanoemulsions has been performed in dendritic cells,^29,41,47,48^ T cells,^40,49^ NK cells,^50^ β cells,^51^ and stem cells.^52–54^ Incubation of the cells with different PFC nanoemulsions did not affect cell viability, making it a viable approach to assess the effective delivery of therapeutic cells to their target.^55–57^ As labelled cells are introduced in a subject, they can be tracked and quantified via MRI and MRS.

With *in situ* cell labelling, PFC nanoemulsions are injected directly into the subject. This approach takes advantage of the phagocytic properties of monocytes and macrophages, where the nanoparticles are small enough to be taken up by these cells. Sites with PFC aggregation, for example, inflammatory sites recruiting labelled macrophages/monocytes, subsequently become visible for 19F MRI, given the sufficient local accumulation of 19F nuclei. Flögel et al. first visualised inflammatory processes *in vivo* using models of cerebral and cardiac ischaemia.^39^ Macrophage tracking has also been performed in numerous disease models.^58–63^ As key players in inflammation, the recruitment of macrophages can serve as a good indication of therapeutic response, whether eventually positive or negative, especially in the context of immunotherapy.^64^ As a disadvantage, nanoprobes can also be taken up/sequestered by other components of the reticuloendothelial system, such as the spleen and the liver, hampering inflammation imaging in these organs.

#### Imaging of hypoxia

Hypoxia as a microenvironmental target has been primarily studied in the context of cancer. However, oximetry of the kidneys, placenta, and brain using 19F MRI has also been demonstrated.^65–68^ In this subsection, we will focus on oncological applications because the majority of 19F pO2 mapping research focuses on that domain.

The tumour microenvironment (TME) is characterised by hypoxia, lower pH levels, altered expression of enzymes, and redox potential, promoting a mutagenic shift leading to malignancy.^69–74^ These alterations at the TME contribute to worsening clinical outcomes by reducing the efficacy of radiotherapy, chemotherapy, and immunotherapy.^75^ Probing TME physiology and its changes is imperative in optimising treatment efficacy and improving patient care. Since tumour oxygen levels are known to be heterogeneous,^32^ imaging-derived oxygen maps could indicate which tumour regions are hypoxic, requiring an adjusted immuno/radiotherapy dose. Due to the linear relationship between the spin-lattice relaxation rate of PFC species and the dissolved oxygen concentration, 19F MRI can generate such maps.^76^ McNab et al. demonstrated the usefulness of 19F hypoxia imaging with their serial scans of on-treatment tumour-bearing mice.^77^ The pO2 values derived from these scans showed that tumour oxygenation differed during the growth, regression, and relapse phases of treatment. In a murine glioblastoma model, increases in pO2, quantified on 19F imaging, were observed at day three post-treatment with CAR-T cells indicative of CAR-T cell homing and tumouricidal activity.^78^ Novel fluorinated contrast agents are emerging that are responsive to hypoxia, potentially providing insight into the dynamic state of oxygenation or serving as theranostics through targeted delivery of cytotoxins.^79–82^

#### pH imaging

Under physiological conditions, the extracellular pH = 7.35–7.45 is tightly regulated. An altered pH balance may be caused by infections, cancer, pulmonary disease, ischemia, and renal diseases.^83–87^ Expanding non-invasive, multiparametric and/or multimodal imaging to probe pathophysiology may support researchers or improve clinical management of these conditions. For example, functional loss often precedes anatomical changes in renal diseases, and early intervention can prevent or attenuate complications.^65^ Electrode-based direct measurement of pH is not only invasive, it also fails to account for heterogeneous pH regions in organs or tumours, which 19F MRI may address.

Studies on pH imaging using 19F MRI are currently limited to *in vitro* conditions, with *in vivo* studies focusing on 19F MR spectroscopy.^88,89^ Nevertheless, research on 19F MRI probes has yielded promising results over the past years. pH-sensitive 19F probes fall within the stimuli-responsive probes category, where changes to the 19F MRI signal become apparent upon encountering the activating condition (OFF/ON). Similar to the design of other environment-responsive probes, alterations to the 19F MR signal may occur via several mechanisms.^31,90–92^

A different approach to 19F MRI pH imaging is with ratiometric pH-sensitive probes, where instead of a OFF/ON signal, the 19F signal scales to the target analyte, allowing pH quantification.^93,94^ Ratiometric probes emit multiple signals in response to analytes such as ion concentration. In the case of NMR, the signal consists of at least two spectral peaks at different chemical shifts which correlate to the analyte concentration. Therefore, the ratio between spectral peaks can be compared to calculate analyte concentrations. Direct pH measurement with ratiometric agents is possible because the 19F MR signal scales proportionally (from one chemical shift to another) within a specific pH range. Janasik et al. proposed a molecular switch system for this purpose. The ratio between the 19F MR peaks of their agent changed over a pH range of 3–4, allowing for sensitive measurements within that range.^94^ Ideally, a pH probe for cancer imaging would be designed to keep its sensitivity within the biological pH range. Janasik et al. recently achieved this through modifications to their molecular switches (pH range 5.5–7), demonstrating the potential of these agents in quantitative 19F MRI.^95^

#### Multimodality imaging

Multimodality imaging combines the power of different imaging techniques to image the same subject. The advantage of such an approach is that one method could compensate for the shortcomings of the other. In the case of 19F MRI, multimodal imaging has been explored with optical fluorescence imaging (OFI), ultrasound sonography, CT, and PET. One of the most prominent examples is the addition of fluorescent dyes to 19F tracers.^55^ Fluorescent probes make it possible to validate the origin of the *in vivo* 19F MRI signal via other methods, such as FACS and OFI. Readers are referred to a recent review by Janasik and Krawczyk for in-depth coverage of multimodality imaging and 19F MRI.^96^

## Section II: Practical considerations for 19F MRI

In this section, we present practical aspects of 19F imaging for beginners and highlight common pitfalls that may occur (with mitigation strategies). The sample protocol, screenshots of common errors, and employed solutions are based on the preclinical MR system at the corresponding author’s centre (Bruker Biospec 7T 70/20). This system is widely used in 19F literature,^54,62,97,98^ and the core information provided can be generalised to other setups.

### Core elements for a 19F MRI study

#### 19F MRI protocols

A standardised protocol helps teams generate images of suitable quality and reproducible characteristics. While 19F MRI resembles conventional methods, some unique aspects apply. Supplementary S1 contains a general 19F imaging procedural protocol that could serve as a starting point for 19F imaging experiments (hosted on protocols.io). Srinivas et al., Waiczies et al., and Hu et al. also provide a step-by-step protocol for quantitative cell tracking *in vivo*, and 19F MRI oximetry.^99–101^

Supplementary S1.

#### Hardware

Due to the similarity of 1H and 19F in MR properties, minimal hardware modifications are required for conventional preclinical imaging systems to perform 19F MRI.^102^ The most significant alteration required is that RF components need to be tuned to the Larmor frequency of the fluorine nucleus. RF coils are essential for creating and detecting an MR signal. They can function as transmitters, receivers, or both (*i.e.,* a transceiver). Hernandez et al. provide a more in-depth review of MR coils.^103^ In addition, readers can consult Table 2, which summarises methods of 19F MRI studies across different applications.

**Table 2.** List of 19F MRI studies with different applications and system specifications

Application	Imaging target (method of labelling or reagent delivery)	Reagent	Sequence (19F MRI)	System	RF Coil	Reference
Imaging of inflammation in tumours	TAMs (*in situ*)	PFCE NE with fluorescent dye	FLASH	7 T BioSpec 70/20 USR; Bruker	custom dual 1H/19 F volume coil, 35 mm diameter	Shin et al. (2017)^97^
Cell tracking after HIFU treatment of tumours	TAMs (*in situ*)	PFCE NE with fluorescent dye	FLASH	7 T BioSpec 70/20 USR; Bruker	custom dual 1H/19 F volume coil, 35 mm diameter	Shin et al. (2018)^45^
Imaging of inflammation in arthritis	Phagocytic cells (*in situ*)	PFCE NE (10% wt/wt)	RARE	7 T BioSpec; Bruker	1H/19F transceiver volume coil, inner diameter of 40 mm (Bruker BioSpin GmbH)	Neveu et al. (2020)^98^
Imaging of inflammation in tumours	TAMs (*in situ*)	VS-1000H; Celsense	RARE (3D)	7 T BioSpec; Bruker	custom dual 1H/19F birdcage coil, 40 mm diameter	Weibel et al. (2013)^62^
Cell tracking	Mesenchymal stromal cells (*ex vivo*)	CS ATM DM Red; Celsense	RARE	7 T BioSpec 70/20 USR; Bruker	dual 1H/19F transceiver linear birdcage, 72 mm inner diameter	Rizzo et al. (2020)^54^
Imaging of inflammation in colitis-associated dysplasia	Macrophages (*in situ*)	VS-1000H DM Red; Celsense	RARE	11.7 T BioSpec 117/16 USR; Bruker	1H/19F tunable 20 mm surface coil	Shin et al. (2017)^61^
Imaging of inflammation in tumours	Macrophages (*in situ*)	VS-1000H DM Red; Celsense	bSSFP	9.4 T small animal MRI system; Varian	custom dual 1H/19F birdcage coil	Makela and Foster (2018)^104^
Imaging of inflammation in tumours	Macrophages (*in situ*)	VS-1000H DM Red; Celsense	bSSFP (3D)	9.4 T small animal MRI system; Varian AND clinical GE 3 T MR750; General Electric	9.4T: custom dual 1H/19F birdcage coil, 30 mm diameter 3T: 43 × 43 mm dual 1H/19F surface coil; Clinical MR Solutions	Makela and Foster (2019)^42^
Imaging of inflammation in tumours	TAMs (*in situ*)	VS-1000H or VS-1000H DM Red; Celsense	RARE	11.7 T BioSpec; Bruker	dual 1H/19F birdcage volume coil; Bruker	Khurana et al. (2018)^44^
Imaging of inflammation in arthritis	Macrophages (*in situ*)	VS-1000H DM Red; Celsense	RARE	7 T Varian DirectiveDrive MRI spectrometer (Agilent technologies)	dual 1H/19F transceiver volume coil, 35 mm inner diameter; m2m Imaging Corp.	Balducci et al. (2012)^58^
Monitoring *in vivo* clearance of NP	RES (*in situ*)	multi core PLGA-PFCE NP	RARE (3D)	11.7 T BioSpec 117/16; Bruker AND Bruker AVANCE III 9.4 T wide‐bore nuclear MR spectrometer; Bruker	dual 1H/19F birdcage coil	Balducci et al. (2012)^60^
Cell tracking	Mesenchymal stromal cells (*ex vivo*)	2.5 mg ml^−1^ PFPE NE Cell Sense; Celsense	bSSFP (3D)	clinical 3 T MRI (Discovery MR750, General Electric)	dual 1H/19F surface coil, 4.31 × 4.31 cm diameter; Clinical MR Solutions	Sehl and Foster (2021)^105^
Cell tracking for drug delivery in Parkinson’s disease models	Transfected macrophages (*ex vivo*)	CS ATM DM Red; Celsense	RARE	unspecified 9.4 T small animal MRI	custom dual 1H/19F volume coil	Haney et al. (2020)^106^
Imaging of inflammation in stroke and ECM bioscaffolds	Phagocytic cells (*in situ*)	VS-1000H DM Red; Celsense	FISP	9.4 T horizontal bore Bruker Avance AV3 HD MR scanner	dual 1H/19F 40 mm coil; Bruker	Modo et al. (2022)^107^
Method optimisation	Phantoms	PFPE NE; Celsense PFCE NE PFOB NE	TSE and bSSFP	3 T Magnetom Prisma clinical scanner; Siemens	dual 1H/19F transceiver birdcage coil; Rapid Biomedical	Colotti et al. (2016)^108^
Method optimisation	Phantoms	Potassium hexafluorophosphate (KPF6)	RARE	7 T BioSpec 70/30 USR; Bruker	dual 1H/19F transceiver linear birdcage RF coil	Mastropietro et al. (2014)^109^
Multimodal theranostics	s.c. KB tumour (i.v. injection)	PLGA-PEG-Folate NP loaded with PFOB, Indocyanine green and Doxorubicin	MSME	16.4 T vertical, wide-bore Bruker Avance III spectrometer; Bruker	not specified	Vu-Quang et al. (2016)^110^
Multispectral imaging	Mononuclear cells from umbilical cord blood (*ex vivo*)	PFCE or PFOB-loaded NP	1.5 T: bSSFP 11.7 T: multislice GRE	1.5 T clinical scanner and 11.7 T Varian scanner	1.5 T: 1H transmit quadrature body coil and receive surface coil, 4 cm diameter. 19F transceiver square surface coil, 5 cm 11.7 T: 1H/19F tunable 3 cm surface coil	Partlow et al. (2007)^111^
Multispectral imaging	Mononuclear cells (*in vivo*)	PFCE and PERFECTA NE	FSE	7 T BioSpec; Bruker	dual 19F/1H transceiver volume coil (35 × 59 mm)	Chirizzi et al. (2019)^112^
Multispectral imaging	TAMs (*in vivo*)	PFCE (10 and 20% wt/vol) and PFTBH NE (60% wt/vol); Aurum Biosciences	TSE and gradient–recalled echo scan	3 T clinical MRI scanner (Prisma, Siemens)	dual 1H/19F transceiver birdcage coil; Rapid Biomedical	Croci et al. (2022)^46^
Microenvironment imaging	Cathepsin K activity	modified FLAME	RARE	11.7 T Bruker BioSpec 117/11; Bruker	dual 1H/19F volume coil, 35 mm inner diameter	Konishi et al. (2021)^113^
Microenvironment imaging	Redox reactions	PFC-encapsulated nanoparticle probe (FLAME-SS-Gd3+)	RARE	Bruker AVANCE III HD 500 and Bruker Avance II 500 WB	unspecified volume coil (25 mm diameter)	Nakamura et al. (2015)^114^
Microenvironment imaging	pH in s.c. 4T1 tumours (i.t. injection)	FNPs-PEG	FLASH	9.4 T BioSpec, Bruker	not specified	Guo et al. (2018)^92^
Microenvironment imaging	pH in s.c. S180 tumour (i.t. injection) MMP-2 activity in HT-1080 xenografts (i.t. injection)	BMMIBF4-pH and BMMIBF4-MMP	FLASH	9.4 T BioSpec 94/20 USR; Bruker	commercially available 1H/19F coil	Zhu et al. (2020)^115^
Cell tracking	Human mesenchymal stem cells (*ex vivo*)	CS-1000 or CS-ATM-DM Red; Celsense	3D bSSFP	9.4 T small animal MRI system; Varian	custom dual 1H/19F birdcage coil, 22 mm i.d.; Agilent	Ribot et al. (2014)^116^
Cell labelling	Therapeutic human tolerogenic DC (*ex vivo*, phantom)	PLGA-PFC NP with fluorescent dye (Cenya Imaging)	2D SSFP	3 T Philips Achieva; Philips Healthcare	19F-tuned surface coil, 20 cm diameter; PulseTeq Ltd.	Cooke et al. (2022)^48^
Imaging of inflammation in atherosclerosis model	Monocytes (*in vivo*)	PFCE NE (10% wt/vol)	TSE	9.4 T horizontal bore animal spectrometer; Varian	custom dual 1H/19F surface coil, 18 mm diameter	van Heeswijk et al. (2015)^59^
Cell tracking	Human neural stem cells (*ex vivo*)	CS-1000; Celsense	TSE	Biospec 11.7 T/16 cm	custom inductively-coupled single-loop surface coils, 9 mm (MRS), 20 mm (*in vivo*), 25 mm (*in vitro*) diameter	Boehm-Sturm et al. (2011)^53^
Microenvironment imaging	Intracellular pO2 (*ex vivo*)	PFCE NE	RARE	11.7 T Bruker BioSpec	dual 1H/19F birdcage volume coil; Bruker	Chapelin et al. (2021)^78^
Microenvironment imaging	Tissue pO2 (i.v. and i.t. injections)	PFCE emulsion (40% wt/vol)	pulse-burst saturation recovery sequence	2.35 T Bruker/SMIS 40 cm horizontal bore imaging spectrometer	unspecified surface coil, 20 mm diameter	McNab et al. (2004)^77^
Cell tracking	Antigen-specific T cells (*ex vivo*)	PFPE NE with fluorescent dye	RARE	11.7 T Bruker	unspecified birdcage coil; Bruker	Srinivas et al. (2009)^43^
Cell tracking	human NK cells (*ex vivo*)	CS-1000 ATM; Celsense	Multi slice spin echo acquisition	Agilent 4.7 T	dual 1H/19F volume quadrature coil	Bouchlaka et al. (2016)^50^
Microenvironment imaging	Intrarenal pO2 (i.v. injection)	PFCE NE (40% vol/vol)	FSE	11.7 T Varian scanner	custom actively decoupled transmit-receive coil pair: 19F transmitter volume coil, 5 cm i.d., and 19F receiver surface coil, 1 × 2 cm	Hu et al. (2014)^65^
Microenvironment imaging	Placental pO2 in preeclampsia model (i.v. injection)	PFCE NE (40% wt/wt) with fluorescent dye	RARE	7 T BioSpec 70/20; Bruker	Dual 1H/19F transceiver volume coil, 40 mm diameter; Bruker	Boehm-Sturm et al. (2021)^67^
Microenvironment imaging	Cerebral pO2 (i.c. injection)	PFCE NE (20% wt/wt) with fluorescent dye	FAIR-EPI	7 T BioSpec; Bruker	Room temperature 1H saddle coil, cryogenic 19F surface coil; Bruker	Khalil et al. (2019)^68^

BMMI, 1-butyl-2,3-dimethyl-imidazolium; DC, dendritic cells; ECM, extracellular matrix; FAIR-EPI, flow-sensitive alternating inversion recovery echo planar imaging; FISP, fast imaging with steady-state free precession; FLAME, Fluorine accumulated silica nanoparticles for MRI contrast enhancement; FLASH, fast low angle shot; FNPs-PEG, 19F MRI nanoprobes based on hybrid metal organic framework core with hydrophilic PEG core; FSE/TSE, fast spin echo/turbo spin echo; GRE, gradient echo; HIFU, high intensity focused ultrasound; MMP-2, matrix metalloproteinase-2; MSME, multi-slice multi-echo; NE, nanoemulsions; NK cells, natural killer cells; NP, nanoparticles; PEG, polyethylene glycol; PERFECTA, super fluorinated contrast agent; PFC, perfluorocarbon; PFCE, perfluoro-15-crown-5 ether; PFOB, perfluorooctyl bromide; PFPE, perfluoropolyether; PFTBH, perfluoro-tert-butylcyclohexane; PLGA, poly(lactic-co-glycolic acid); RARE, rapid acquisition with relaxation enhancement; RES, reticuloendothelial system; RF, radiofrequency; SSFP, steady-state free precession; TAMs, tumour-associated macrophages; bSSFP, balanced steady-state free precession; i.c., intracerebral; i.t., intratumoural; i.v., intravenous; s.c., subcutaneous.

The two most common coil geometry designs are volume and surface coils. Volume coils are used for whole sample imaging as the transmitted B_1_ magnetic field is more homogenous and extends over a larger area. This characteristic is advantageous in 19F applications that require a uniform signal strength, for example, quantifying *in vivo* 19F signal with an external reference. Surface coils, on the other hand, offer more sensitivity than volume coils. However, their B_1_ field decays further away from the coil and is not uniformly distributed, resulting in a small sensitivity volume and imaging penetration depth. Despite these disadvantages, surface coils offer benefits in 19F MRI when acquiring images of structures close to the coil’s surface.^117^

When considering the RF coil design, how a coil is tuned to the correct nucleus is also essential. Single- and dual-tuned coils have been used in 19F MRI studies, with 19F quadrature coils having a superior sensitivity than linear coils. However, a linear 1H/19F dual-frequency volume coil provides more convenience. Since the 1H/19F coil does not have to be switched after performing system adjustments and acquiring 1H MR images, the general workflow and co-localisation of the 1H and 19F MR signal are improved. Proton-based B_0_ mapping can be used to correct for static magnetic field inhomogeneities via shim currents, and inaccurate co-registration of the 1H and 19F images is prevented. Furthermore, optimal RF power settings and B_1_ profiles can be derived from the 1H signal. They can be used to determine parameters for the fluorine nucleus, while surface coils may require a 19F reference.^118^ Dual-frequency coils can also be designed to allow for simultaneous 1H/19F MRI improving co-registration^119–121^ but would then require additional hardware modifications.

Coil construction materials influence coil system choice as they may impact image quality. For example, proton-bearing materials used in MRI hardware equipment, such as polycarbonate, contribute to the background signal in 1H MRI.^122^ Fluorinated materials such as polytetrafluorethylene (Teflon) and fluorinated oils are commonly used in commercial and custom-built MRI equipment.^123^ While these are beneficial in proton imaging, they form a possible source of background in 19F MR studies.^100^

#### Sequences

Pulse sequence choice for a given 19F MRI study requires careful consideration to improve the sensitivity. The complexity of an agent’s MR spectra, chemical shift, and relaxation properties influences optimal signal detection. As these vary from agent to agent, there is no universal pulse sequence with optimal performance. The high degree of freedom and complexity in crafting a 19F sequence is reflected in the multiple works on optimising 19F imaging strategies to improve sensitivity.^26,108,109,124–126^ Table 3 lists sequences used for various applications of 19F MRI. In addition, the specific absorption rate (SAR), a metric of the amount of RF energy deposited in tissues generated by any given sequence, should be kept in mind during experimental design. Due to the inherent low sensitivity of 19F MRI, studies benefit from fast imaging sequences, which apply multiple RF pulses in rapid succession (*e.g.,* SSFP, UTE, and ZTE). The application of such sequences poses a challenge in preclinical and clinical applications where SAR is limited due to safety.

**Table 3.** Overview of commonly used pulse sequences in 19F MRI

Sequence	Probes	Advantages	Disadvantages
RARE	Compatible with probes with long T2	Less susceptible to B_0_ inhomogeneities	When using a high echo train length, watch the specific absorption rate
		Increase echo train length to shorten acquisition time	
		Best for imaging larger regions with PFCs and a large number of slices	
3D RARE		3D RARE has excellent SNR efficiency	Longer absolute acquisition time (3D)
FLASH	-	Possibility to use low flip angles	Low SNR efficiency
bSSFP	Performs best with strong T2/T1 ratio	Most SNR efficiency	Off-resonant effects at high B_0_ cause signal loss and worsening of banding artefacts
FISP	-	High SNR efficiency when imaging small areas	Susceptible to flow and motion
UTE	19F agents with ultrashort T2	High SNR due to capturing the signal before line dephasing.	High specific absorption rate
		Both 2D and 3D imaging are possible.	Artefacts may arise due to non-cartesian k-space sampling
			The gradient trajectory of the read gradient must be acquired a priori, which is difficult if agents have a low 19F content
ZTE	19F agents with ultrashort T2	Silent MRI	No slice selection, only possible as 3D sequence
	Agents with lower 19F content	More convenient than UTE: no gradient trajectory measurement required	High specific absorption rate
			Shading artefacts
UTE-bSSFP	Multiresonant agents (*e.g.,* PFOB)	Samples multiple peaks of agents with a complex spectra, improving sensitivity to these agents	Limited applicability as 3D radial sampling and FID readout decrease SNR efficiency in agents with a single resonance peak
			Due to bandwidth restrictions only resonance peaks can be detected within proximity of 1–2 kHz to each other
			Banding artefacts
FREDOM: Pulse burst saturation recovery (PBSR) echo planar imaging (EPI)	pO2 sensitive probes (*e.g.,* HFB)	Quantitative oximetry	FREDOM is limited to easily accessible tumours as it requires intratumoural injection of the 19F probe

FID, free induction decay; FISP, fast imaging with steady-state free precession; FLASH, fast low angle shot; FREDOM, fluorocarbon relaxometry using echo planar imaging for dynamic oxygen mapping; HFB, hexafluorobenzene; PFC, perfluorocarbon; PFOB, perfluorooctyl bromide; RARE, rapid acquisition with relaxation enhancement; UTE, ultrashort echo time; ZTE, zero echo time; bSSFP, balanced steady-state free precession.

#### 19F MRI agents

Several 19F MRI agents exist, with PFCs being the most commonly used (Table 4). Agent design and formulation of PFCs and other agents are still under development, intending to improve sensitivity, delivery, function and clearance (Table 5). Nanocarriers improve the solubility and delivery of PFCs and influence the clearance rate.^150^ Nanoparticles and nanogels have the added benefit of being stable as opposed to lipid-based nanoemulsions, which have a relatively short shelf-life due to Ostwald ripening. A nanocarrier’s structure can be modified to confer new characteristics (*i.e.,* functionalisation), enabling cell targeting, reactivity to stimuli, and multimodal imaging. One of the earliest methods of 19F probe functionalisation was incorporating fluorescent dyes in PFC nanoemulsions.^55^ The added fluorescent moiety allowed for the *ex vivo* validation of labelled cells with microscopy and flow cytometry. Other nanocarriers have been developed to expand the functionality of 19F MRI agents.^135,149,151,152^

**Table 4.** Properties of different classes of fluorinated agents used in 19F MRI

Class	Name	Molecular formula	Molecular weight (g/mol)	NMR spectrum	Chemical shift with respect to [reference]	Remarks	Reference
PFC	perfluoro-15-crown-5 ether (PFCE)	C_10_F_20_O_5_	580	simple	−91.8 ppm [CFCl3]	Widely used in cell tracking studies due to its simple NMR spectrum and high number of chemically equivalent 19F atoms. PFCE nanoemulsions have a long biological half-life.	Jacoby et al. (2014)^127^
	perfluorooctyl bromide (PFOB)	CF_3_-(CF_2_)_6_-CF_2_Br	498.96	complex	Total of eight peaks (for each CF_n_ moiety)	Used as a blood substitute and for cell tracking. High potential for clinical translation due to short biological half-life.	Barnett et al. (2011)^128^
	PERFECTA	C_21_H_8_F_36_O_4_	1008.27	simple	−73.48 ppm [TFE]	Used for cell tracking. Has 36 chemically equivalent 19F atoms.	Chirizzi et al. (2019), Tirotta et al. (2014)^112,129^
	perfluorodecalin (PFD)	C_10_F_18_	462.08	complex	Total of ten distinct fluorinated groups resulting in five cis- and trans-isomer pairs. Chemical shifts of pairs one to four are in the range of −115 to −140 ppm and of pair five at −190 ppm [TFA]	Used as an oxygen probe and for cell tracking.	Gulyaev et al. (2019)^130^
	hexafluorobenzene (HFB)	C_6_F_6_	186.05	simple	−164.9 ppm	Used as an oxygen probe. Displayed tissue toxicity after intramuscular administration in a tumour model.	Mignion et al. (2013)^131^
Polymers	perfluoropolyether (PFPE)	CF_3_O[CF_2_O]_n_COF	1380 on average, depends on chain length	complex	major peak at −91 ppm and minor peaks at −58 and −93 ppm [CFCl3]	A polymer of varying chain length with a functionisable terminal group. Widely used in cell tracking studies due to its high fluorine content. A previously commercially available nanoemulsion formulation was tested in a clinical trial. PFPE nanoemulsions have a long biological half-life.	Kadayakkara et al. (2014), Ahrens et al. (2014)^132,133^
	poly(OEGA)m-PFPE	-	3180 (Mn) - 6580 (Mn), depending on OEGA-repeats	complex	major peak ~−80 ppm	PFPE-based co-polymer with lipophilic and hydrophilic subunits leading to reduced macrophage uptake and adjustable blood circulation time.	Zhang et al. (2017)^134^
Branched polymers	Branched Fluorinated Glycopolymers	-	2,8100 (Mw branched polymer 1) and 8250 (Mw linear polymer)	simple	−74.1 ppm [TMS]	Contrast agents with varying fluorine content and designed to target cancer cells. The branches contain a disulfide-bond which is cleaved under reducing conditions resulting in linear polymers and increased 19F MR signal.	Fu et al. (2019)^135^
	PDMAEA-stat-tFEA	-	12.2 (Mn for polymer 1)	simple	−73 ppm [CFCl3]	Hyperbranched polymers for cell targeting with long T2. The agent is cleared via the kidneys.	Thurecht et al. (2010)^136^
	PEG-OMe-PIMA-PEG600-CF4	-	-	simple	~ −62 ppm	Water-compatible fluorine-rich polymers. A copolymer with PEG blocks linking -CF3 groups to its molecular backbone.	Du et al. (2022)^137^
Dendrimers	19FIT-27	-	1908	simple	~ −74.5 ppm [CFCl3]	A water-soluble asymmetric dendrimer carrying 27 equivalent 19F atoms. 19FIT-27 has been used for whole-body dynamic 19F MRI to study organ kinetics. Metabolic stability and fast clearance have been shown.	Jiang and Yu (2010), Liu et al. (2019)^138,139^
	Amphiphile 11	C_510_H_897_F_48_N_25_O_224_	11868.9		−74.49 [CFCl3]	Dual-modal 19F MRI/fluorescence dendrimer carrying 48 chemically equivalent fluorines.	Bo et al. (2015)^140^
	PAMAM-g-PTFPMA-co-PMANa	-	66,6000 (Mn)	complex	−124 and −138 ppm [TFT]	Water-soluble fluorinated polymer nanoparticle which has been tested *in vivo*.	Ogawa et al. (2012)^141^
Fluorinated ionic liquids	tetrafluoroborate	BF_4_^-^	86.81	simple	−150 ppm	Fluorinated ionic liquids are composed of molten salts, which are entrapped in mesoporous silica nanoparticles with stimuli-responsive properties.	Zhu et al. (2022)^142^
	difluoroacetate	C_2_HF_2_O_2_^-^	95.02	simple	−125 ppm		
	trifluoromethanesulfonate	C_3_O_3_S^-^	149.07	simple	−78.8 ppm		

19FIT, 19F imaging tracer; Mn, number average molecular weight; Mw, weight average molecular weight; OEGA, oligo(ethylene glycol) methyl ether acrylate; PAMAM, poly(amidoamine); PDMAEMA, poly((N,N-dimethylamino)ethyl methacrylate); PEG, polyethylene glycol; PEGMA, polyethyleneglycol monomethylether methacrylate; PERFECTA, super fluorinated contrast agent; PIMA, poly(isobutylene-alt-maleic anhydride); PMANa, sodium polymethacrylate; PTFPMA, poly(2,2,3,3-tetrafluoropropyl methacrylate); TFA, trifluoroacetic acid; TFE, trifluoroethanol; TFT, α,α,α-trifluorotoluene; ppm, parts per million; tFEA, trifluoroethylacrylate.

**Table 5.** Selected examples of nanocarrier formulations for 19F MRI

Formulations	Description	Examples	Characteristics	Reference
Nano emulsions (NE)	Oil-in-water NE which are stabilised with a surfactant to improve PFC solubility. Surfactants such as phospholipids and pluronics are required to stabilise NE.	fluorescent blended PFPE amide and PFPE NE	Covalently-conjugated fluorescent dye to the PFC prevents separation of dye from PFC core after NE uptake	Janjic et al. (2008)^55^
		Click-Ready PFC NE	Functionalization of the NE surface is done post-emulsification allowing for the incorporation of more fragile ligands (*e.g.* proteins)	Perez et al. (2022)^143^
Nanoparticles (NP)	Biocompatible particles containing a fluorous core encapsulated by a silica shell. Silica nanoparticles have modifiable internal and external surfaces to which functional groups can be attached.	FLAME	Amorphous silica shell with improved stability and solubility	Matsushita et al. (2014)^144^
			Use of paramagnetic Gd3+ to create stimuli-responsive OFF/ON NPs	Nakamura et al. (2015), Konishi et al. (2021)^113,114^
			Controlled biodistribution via surface modifications shown in multispectral MRI study	Akazawa et al. (2018)^145^
		mFLAME	Mesoporous silica shell with controllable pore size for theranostics: Drug delivery, surface modifiability and dual modal imaging	Nakamura et al. (2015)^146^
	PLGA - Polymer-based nanoparticle system based on poly(lactic-co-glycolic acid). May be loaded with PFC multi-cores.	PFCE PLGA NP	Multi-core structure for fast clearance	Hoogendijk et al. (2020)^147^
			GMP-grade scale up possible	
		PERFECTA PLGA NP	Faster imaging possible due to shortened T1 relaxation	Chirizzi et al. (2022)^148^
Nanogels	Nanoscale hydrogels consisting of a fluorinated polymer-based network	Polymer nanogel P3H	Increased T2 relaxation through post-assembly modifications for improved signal detection	Munkhbat et al. (2019)^149^
		pH-sensitive PEGylated nanogels	OFF/ON switchable probe for pH detection based on volume-phase transition	Oishi et al. (2007)^91^

GMP, Good Manufacturing Practices; PEG, polyethylene glycol; PERFECTA, super fluorinated contrast agent; PFC, perfluorocarbon; PFCE, perfluoro-15-crown-5 ether; PFPE, perfluoropolyether; PLGA, poly(lactic-co-glycolic acid); (m)FLAME, (mesoporous) fluorine accumulated silica nanoparticles for MRI contrast enhancement.

Clearance of PFC nanoemulsions occurs via emulsion breakdown, the release of PFCs into the bloodstream, and subsequent exhalation.^60^ The biological half-life of PFCs can range from 3 to 8 days (*e.g.,* perfluorooctyl bromide) to well over 100 days (*e.g.,* PFCE) in mice.^153^ While shorter biological half-lives are preferred for clinical translation, PFCs such as PFCE remain attractive agents in research due to highly advantageous 19F MRI properties (*i.e.,* simple NMR spectrum and high 19F payload per molecule).^60,154^ Specific modifications to the architecture of a nanocarrier have been shown to enhance the clearance significantly (up to 15x faster), bringing this technology one step closer to the clinic.^60^

19F MRI agents are not yet widely available commercially, and their manufacturing process requires specialised equipment and expertise to guarantee particle uniformity and stability.^55,56^ For medical imaging, multimodal polymeric PFC nanoparticles have been an emerging focus of development.^56,155^ The polymer in these particles, poly(lactic-co-glycolic acid) or PLGA, is widely used in drug delivery and can be functionalised to expand its applications.^156^ To bring PLGA-PFC nanoparticles closer to the clinic, methods for the scale-up of GMP-grade production have been developed.^147,155^

### Common pitfalls in 19f MRI

The primary purpose of this section is to prevent common beginner mistakes we encountered during our first 19F MRI studies. Something as simple as changing the coil operation to the correct nucleus may be overlooked during a lengthy phantom acquisition at the expense of time and effort. 19F MRI is not only hampered by low sensitivity. Similar to 1H MRI, artefacts can be present in 19F images, such as chemical shift displacement, distortions, and banding. Proper mitigation strategies can address many of the artefacts encountered.

#### Sub-optimal transmitter gain adjustment

**Figure 2.**
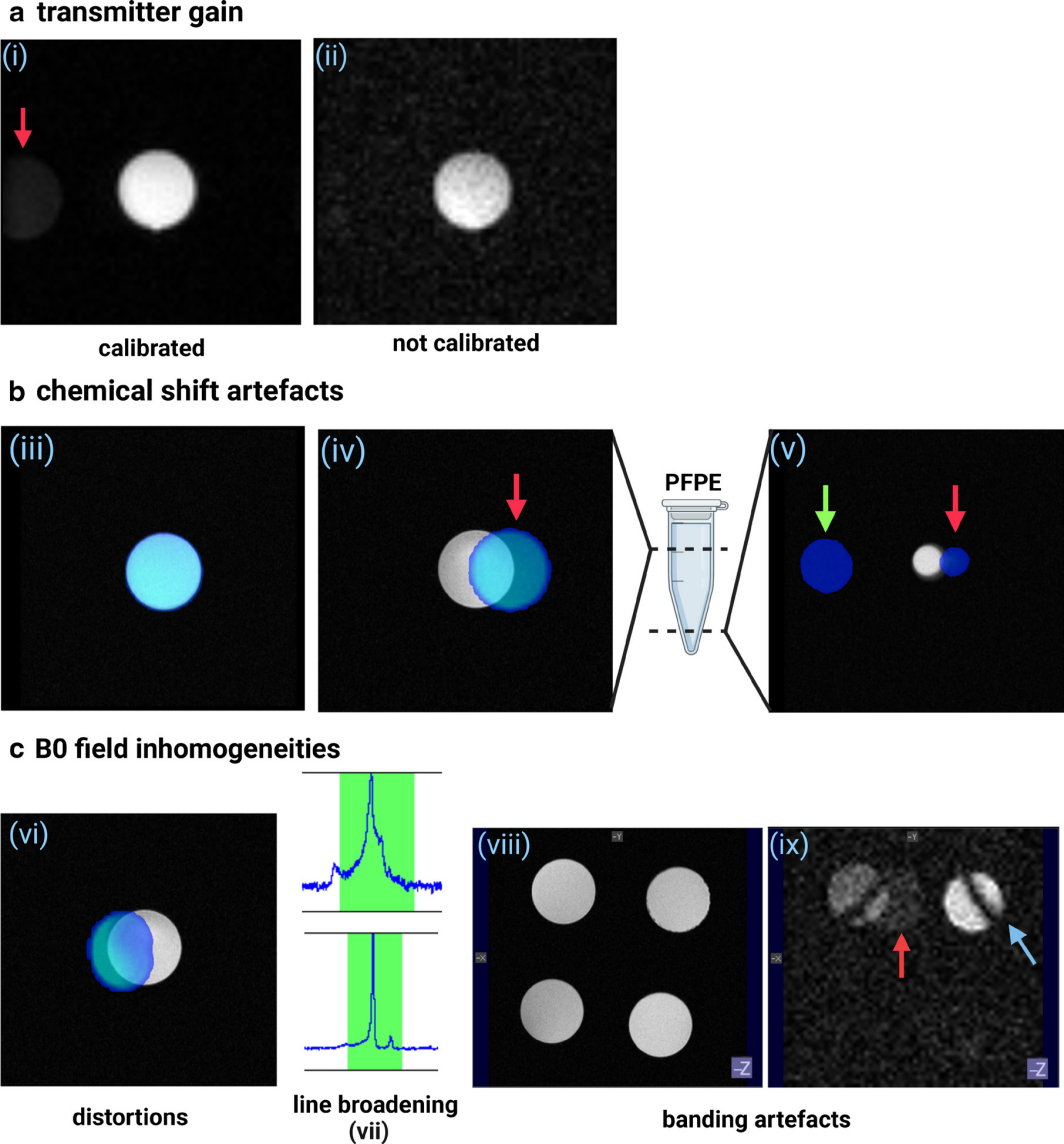
Pitfalls in 19F MRI for beginners. 1H and 19F MR images were acquired in PFPE phantoms at 7T. 1H/19F images are depicted in greyscale (1H) and pseudocolour (19F). Image (iii) is the correct example. (**a**) Influence of transmitter gain calibrations on image quality, comparing 19F FSE acquisitions of (i) calibrated and (ii) uncalibrated RF pulse power. (**b**) and (**c**) Examples of chemical shift artefacts along the directions of frequency-encoding (iv) (v) and (ix), and slice selection (v) indicated by the red and green arrows, respectively. 19F images are superimposed in pseudocolour on top of the 1H images to illustrate the mismapping of the MR signal when selecting an off-resonance working frequency. PFPE has a major and a minor resonance peak in close proximity to each other. NMR signals from the minor peak are not detected at *in vivo* tracer concentrations. The chemical shift artefact recorded at a different slice (iv, green arrow) occurred due to imaging with an undiluted PFPE phantom. (**c**) Main magnetic field (B_0_) inhomogeneities influence the Larmor frequency of 19F spins, causing distortions (vi), loss of signal due to line broadening (vii), and banding artefacts (ix). The 19F MR image in pseudocolour (v) was acquired with a fast spin echo sequence using erroneous shim values, while the 1H acquisition (greyscale) was acquired with proper shims. The 19F NMR spectrum of PFPE depicted in the upper graph was acquired in an inhomogeneous B_0_ field and displays line broadening and increased noise compared to the bottom graph, which was acquired in a shimmed system. 1H FSE (viii) and bSSFP 19F (ix) MRI of four tubes filled with serially diluted PFPE in 1% low-melting point agarose. The bSSFP sequence is sensitive to B_0_ inhomogeneities leading to the appearance of banding artefacts (blue arrow). Tissue-border interactions (in this case, the four tubes and air) perturb B_0_ homogeneity, which may be challenging to fully correct via shimming. The four tubes with PFPE could instead be embedded within another tube with agarose to minimise tissue-border interactions. The bottom tubes were below the detection limits and, therefore, not visible in the 19F image. bSSFP: balanced steady-state free precession; FSE: fast spin echo; PFPE: perfluoropolyether; RF: radiofrequency

In order to excite a spin system, an RF pulse is required. The required power for such a pulse depends on several factors, including sample load. In conventional MRI, system software automatically calibrates pulse amplitude before image acquisition to determine the required power for a 90 degree RF pulse. This automated approach is not possible in 19F MRI because the 19F MR signal is too low, requiring the reference power to be set manually. In the case of a dual-frequency 1H/19F coil, reference power values can be derived from the 1H channel using even non-fluorinated subjects. However, single-frequency 19F coils require an external, concentrated 19F reference with sufficient signal to calibrate the reference power. Under optimal acquisition conditions, the 19F image generated demonstrates the maximum possible SNR (given the sample, Figure 2a [i]). When the reference power is below the required value, the SNR is negatively impacted, yielding a significantly noisier image (Figure 2a, [ii]). To prevent or overcome this issue, the transmitter gain, and other system adjustments, can be manually determined using a concentrated 19F reference standard to guarantee optimal adjustments.^99^

#### Chemical shift artefacts

Every 19F contrast agent displays a unique spectral profile, where the intensity of the fluorine signal is greatest at a particular chemical shift (expressed in ppm). At the start of the image acquisition process, users need to define an operating frequency instructing the scanner to excite a specific ppm range. Under normal circumstances, when the peak is correctly identified for the given fluorinated compound, the 19F image and underlying anatomical image should have near-perfect co-localisation (Figure 2b, [iii]). When the peak is incorrectly defined due to a human mistake or an underlying change in the nature of the compound (*e.g.,* degradation), a chemical shift artefact occurs where the 19F signal detected by the coil is mapped out to incorrect coordinates in space. This error leads to a 19F signal being recorded elsewhere in the image, which can be in the frequency encoding or slice selection direction (Figure 2).

Imaging with multiple 19F agents or agents with a complex spectral profile may also be a source of chemical shift artefacts due to magnetically non-equivalent 19F spins. A way to circumvent this is via chemical presaturation RF pulses (*e.g.,* fat-sat in 1H MRI).^157^ These pulses excite spins within a selected chemical shift responsible for the artefacts. Chemical saturation is performed before the image acquisition, thereby suppressing the MR signal during subsequent MRI. Another way to circumvent chemical shift artefacts is through selective excitation of a (subset of) spectral line(s) at the cost of SNR.^158^ The sparse nature of the 19F signal *in vivo* would benefit from specialised imaging approaches that record the MR signal from all spins without the artefacts. Goette et al. described an acquisition strategy that acquires the 19F signal of perfluorooctyl bromide (PFOB) before de-phasing spins using balanced UTE-SSFP and free induction decay readout.^159^ Additionally, van Heeswijk et al. proposed a method to image 19F agents with complex spectra, where a series of multiecho acquisitions and image reconstructions via a model remove the artefacts.^160^

#### Isoflurane artefacts

Isoflurane is a commonly used volatile anaesthetic in rodents. At therapeutic concentrations and over long timespans, isoflurane is detectable in MRI, which may lead to artefacts. This compound accumulates in the skeletal muscle and fat of the thorax.^161^ As such, the accumulated fluorine signal is picked up by the radiofrequency coil. Fluorine signal from the anaesthetic mixes with the signal from the target compound, yielding false-positive “hot spots” and making quantification more challenging. Staal et al. devised several strategies to address isoflurane artefacts: suppression pulse, out-of-plane shift, and for 3D imaging, narrow excitation bandwidth.^162^ Alternatively, non-fluorinated injection anaesthetics may be considered, such as ketamine with xylazine. Still, this combination may be less suitable for shorter procedures, as the recovery time is significantly longer.^163^

#### Distortions in the main magnetic field

The main magnetic field B_0_ is prone to distortions, which are aggravated at higher field strengths. As such, B_0_ inhomogeneities must be corrected prior to image acquisition to improve image quality. Magnetic field corrections are carried out via magnetic shimming, which superimposes a correction field in order to homogenise the B_0_ field distribution. Suboptimal shimming may lead to peak-broadening, image distortions and artefacts, such as banding artefacts in bSSFP sequences (Figure 2c). The MRI system can perform iterative shimming automatically for low-order gradients, which show the greatest variation.^164^ In addition, other sequences and suppression strategies require high-order B_0_ corrections that must be preceded by quantifying B_0_ field distribution (B_0_ mapping).^165^

#### Cell number quantification

One of the core selling points of 19F MRI is the direct quantitative nature of fluorinated agents imaged in a background-free setting. Quantitative 19F MRI of *ex vivo* or *in situ* labelled cells requires additional considerations, addressed in detail in Srinivas et al.^17,40^

Attempts at accurate cell number quantifications might be hindered due to larger voxel sizes in 19F MRI. For a given image, a voxel represents the average signal within the predetermined volume (*e.g.,* voxel dimensions), encoding the 19F signal from multiple labelled cells within these dimensions. The voxel volume in 19F MRI is relatively large, meaning the lower spatial resolution leads to the loss of fine data on cell location and quantity. Two voxels with the same signal intensity could be derived from cell clusters in different spatial patterns (*e.g.,* clustered *vs* diffuse). Hence, a positive 19F signal might not give the full spatiotemporal context of the region of interest. These challenges might be mitigated using multimodal agents (mentioned above), where the 19F signal could be correlated, for instance, with microscopy for co-localisation.

Cell number quantification can also be hampered due to low sensitivity through the requirement of a higher cell detection limit (*e.g.,* a minimal “critical mass” of 19F-labelled cells to be detected by the RF coil). The cell detection limit is influenced by 19F contrast agent uptake by the labelled cells, the magnetic field strength, hardware, type of tracer, pulse sequence, and imaging time.^27^ Higher cell detection limits require a larger number of cells to accumulate within the voxel dimensions to generate a positive 19F signal. Thus, any signal from a cluster of 19F-labelled cells below this threshold cannot be distinguished from noise while possibly being biologically relevant.

Various cell detection limits have been reported in 19F MRI. Srinivas et al. (2012) compared the detection limit of different PFC-based cell tracking studies at high and ultra-high field strengths by harmonising field strength, SNR, and imaging time.^27^ Numbers as low as 200 and 1000 cells/voxel have been reported with *in vitro* imaged macrophages and neural stem cells, respectively.^39,53^ However, detection limits in the range of 10^3^ to 10^4^ cells/voxel are more typical *in vivo*.^40^ At clinical field strengths, the detection limit for different cell pellets ranged between 2.5*10^4^ macrophages/voxel (SNR = 5)^42^ to 1.0*10^5^ DCs/voxel (SNR = 2.5).^132^ *In vivo*, the sensitivity was reported at 10^6^–10^7^ human DCs/voxel (SNR = 2.5).^132^ While the cell detection limits may vary across studies and are difficult to compare, having a general idea of the limitations of your own system is crucial in the preparation of 19F MRI studies. This can be achieved using phantoms of cell suspensions at different concentrations with known labelling efficiency.^166^

Possible causes for mis-quantification include non-uniformity of the B_1_ field, partial volume effects, and tracer loss (*e.g.,* cell division or apoptosis).^125,167^ The homogeneity of the B_1_ field depends on different factors, including coil and sample geometry. Detailed explanations on how to carry out B_1_ corrections in 19F MRI are discussed by Vernikouskaya et al. and Srinivas et al., and in a cryogenically cooled RF coil by Delgado et al..^99,168,169^

## Section III: Future perspectives and conclusions

### Future perspectives

#### Addressing sensitivity challenges

Improved hardware, sequences, and novel 19F probes have given rise to novel sensitivity-boosting strategies such as compressed sensing, paramagnetic agents, branched systems, and cryo-cooling technology.^68,170–174^

In compressed sensing, k-space data are undersampled and reconstructed, reducing imaging time while denoising the acquired image.^175–179^ Recently, Chen and colleagues combined compressed sensing with ZTE, resulting in a pulse sequence suitable for fluorinated probes with short T1 and T2 such as PFCs with a metal chelate. Applying a compressed sensing ZTE sequence together with a metallo-PFC probe significantly reduces the acquisition time of isotropic 3D images.^172^

The sensitivity of 19F MRI can be further enhanced through alterations to the MR properties of 19F agents. PFCs typically display long T1, reducing the possible number of excitations within a fixed time.^180^ Scan times can be reduced significantly by exploiting paramagnetic metals.^26,171,181^ The T1 of 19F agents decreases in the presence of lanthanides(III) or iron(II) atoms^182^ due to paramagnetic relaxation enhancement. This effect may also alter an agent’s T2, as seen in some OFF/ON switchable probes discussed previously. Therefore, careful probe design is required to balance effective T1 reduction while maintaining an optimal T2.^183^ Branched systems are another option to increase the sensitivity of 19F MRI due to high 19F payload.^173^ The advent of novel 19F tracers together with novel insights on probe clearance will bring 19F MRI closer to the clinic.^60^

Transceiver surface RF coils may become more common in MRI due to advancements in cryogenically cooled RF probe development.^169^ This technology has been deployed in X-nuclei imaging to reduce thermal noise and improve the detection sensitivity of the coil. Waiczies et al. were the first to apply this for 19F MRI using a transceive quadrature surface coil, improving both SNR and resolution.^174^ The SNR gain can also be interchanged for reduced acquisition time, a most welcome benefit in 19F MRI studies. This method was further improved through B_1_ inhomogeneity corrections and set-up adjustments allowing for quantitative 19F MRI *in vivo*.^169^

#### Multispectral imaging

**Figure 3.**
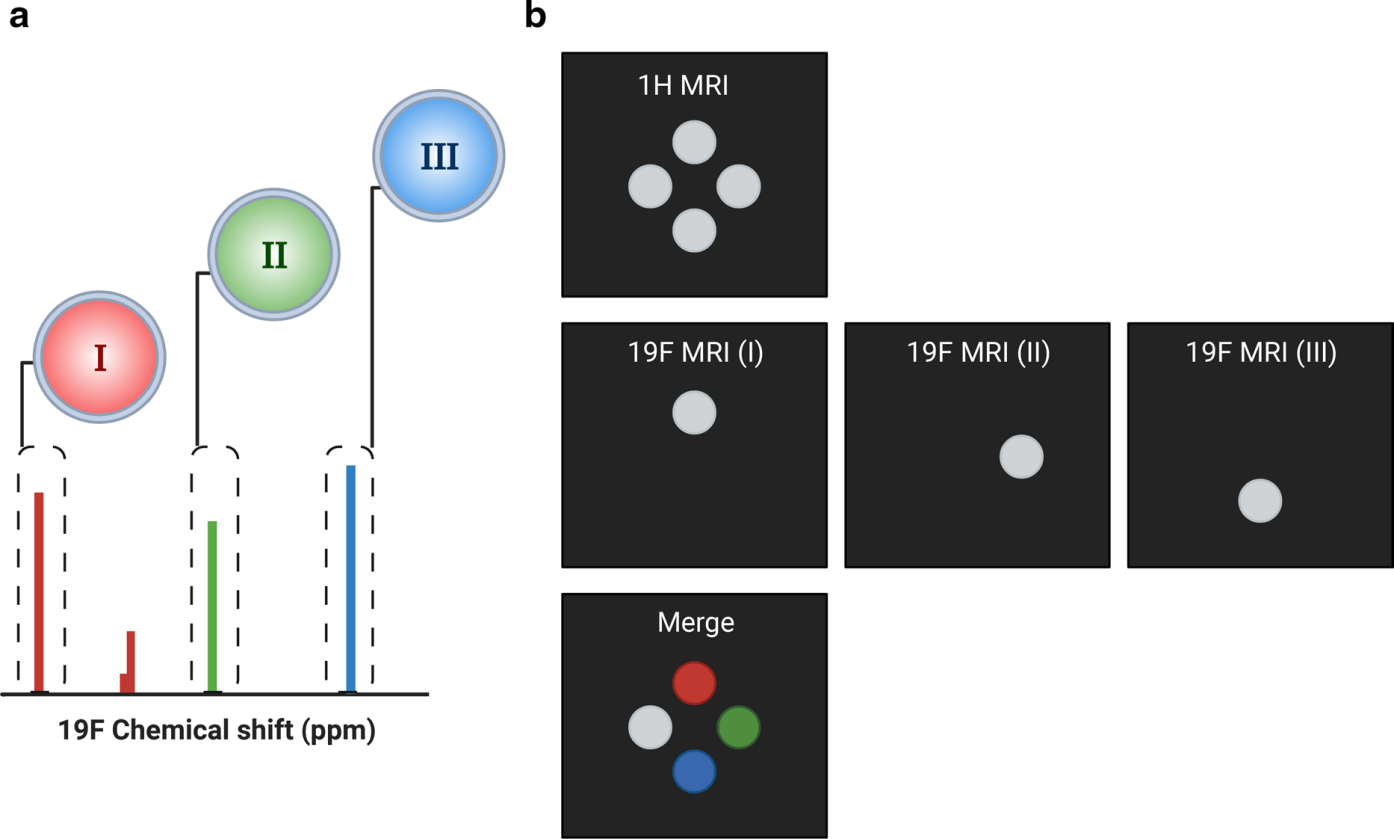
Basic principle of multispectral 19F MRI (**a**) Schematic representation of NMR spectra of three different fluorinated nanoprobes: I, II and III, each with distinct chemical shifts. The dashed boxes enclose the spectral peaks selected for 19F MRI. (**b**) Schematic representation of multispectral 19F MRI with phantoms. From top to bottom: 1H MRI for anatomical context, 19F MRI with the working frequency set to the respective nanoprobe (I, II or III) and a merged image with the 19F signals in pseudo-colour.

Multispectral 19F MRI, also known as multicolour MRI, has gained prominence, which allows the registration of multiple 19F probes within a single subject (Figure 1).^111^ Due to the broad chemical shift range of 19F imaging agents, different probes can be administered simultaneously and detected independently of each other (Figure 3a).^184^ This is also possible with overlapping spectra.^185^ Conversely, the number of tracers is limited in PET and SPECT. Different 19F probes receive their own ‘colour’ after merging all the signals and placing them within anatomical context (Figure 3b).

The need to track different cell types provided the motivation to explore multispectral 19F MRI. Partlow et al. used PFCE- and PFOB-loaded nanoparticles to *ex vivo* label mononuclear cells and subsequently visualise them *in situ*.^111^ Since then, multiple multispectral 19F MRI studies have followed. Chirizzi et al. used multispectral imaging to monitor changes in phagocytic activity of mononuclear cells induced after immune modulation.^112^ Multicolor MRI has also been used to study the behaviour of silica- and PLGA-based nanoparticle formulations *in vivo*.^57,145^ Through characterisation of the biodistribution and degradation patterns of different nanocarrier systems, researchers may select a formulation compatible with a particular application.

Multispectral 19F MRI has been expanded to other domains of personalised medicine, and it is an exciting development within the field. For cancer imaging, the ideal setting would be to identify cell populations which may negatively impact treatment, such as myeloid-derived suppressor cells, regulatory T cells, and pro-cancerous tumour-associated macrophages (TAMs). Through multispectral 19F MRI, Croci et al. were able to investigate the spatio-temporal dynamics of TAMs in high-grade gliomas upon treatment and recurrence.^46^ They could map the abundance and location of distinct TAM niches, either originating from tissue-resident glial cells or monocyte-derived macrophages, as well as detect phenotypic changes with additional methods. Beyond population dynamics, multispectral MRI can be used to probe other factors at the TME. Mesoporous silica nanoparticles loaded with fluorinated ionic liquids were used for multicolour, stimuli-responsive imaging in tumours.^142^ Another remarkable achievement has been capturing the development of cardiovascular disease.^186^ Flögel and colleagues functionalised PFC nanoemulsions with peptide ligands and single-chain antibodies that could target proteins and inflammation.^186^ Most strikingly, this study highlights the potential of multispectral MRI to identify imaging biomarkers of vascular inflammation well before the manifestation of disease.^186^ Multiplex approaches described above may form one of the stepping stones towards implementing molecular MRI in the diagnosis, prognosis, and monitoring of complex diseases.

#### 19F MRI in humans

Due to the sensitivity challenges in 19F MRI, high-field MRI setups with specially designed coils are essential. Unfortunately, such equipment is not readily available in many healthcare centres, where MR scanners operate at far lower field strengths. The technical obstacles and limited toxicological characterisation of novel 19F agents have resulted in 19F molecular imaging being primarily studied in preclinical settings. However, a small cohort of innovative studies has found workaround solutions to some of these challenges, and 19F MR scans in humans have been successfully performed.

Lung imaging is currently the most frequent use-case of 19F MRI in the clinic, although uncommon. Functional 1H MRI of the lung is challenging and can be improved using hyperpolarised gases. Alternatively, lung function can be assessed via the inhalation of inert-fluorinated gases and 19F MRI, with the advantage that it can be administered alongside O2 without a detrimental effect to image quality and does not require a polariser.^187^ In humans, small feasibility studies of 19F MRI in the lung have been performed at varying field strengths (0.5–3T).^188–192^ Developments in guided reconstruction of undersampled 19F MR images have been reported, reducing the imaging time.^193^ This improves clinical translatability as patients with lung diseases such as chronic obstructive pulmonary disease experience discomfort and may not attain the length of a breathhold required for image acquisition.

As the new generation of stimuli-responsive and targeted probes are still being explored as proof-of-concepts, cell labelling in humans has only been carried out once using a PFPE nanoemulsion. In 2014, a small clinical trial was performed to test DC tracking at 3T in stage IV colorectal cancer patients who received an intradermal DC injection.^132^ The PFPE-labelled DCs were visualised at multiple time points post-injection using a surface coil. This was successful at the injection site; however, cell tracking was unattainable due to cell detection limits. Despite this limitation, 19F cell labelling remains an attractive strategy to visualise and track therapeutic cells, potentially in humans.^47,48^ As 7T MR scanners are being introduced into clinical practice and more expertise is built in human high-field MR imaging, we expect that 19F clinical implementation will be a topic that will be revisited in the literature.

## Conclusions

19F MRI is a promising modality for targeted/molecular imaging. While the technique has existed for decades, advances in (ultra-)high field magnetic resonance, contrast agent synthesis, and pulse sequence development have allowed proof-of-concept studies for various use cases. Its unique properties of being non-radioactive, highly specific and sensitive to the local environment make it a potentially valuable tool for gaining non-invasive insight into the biology of multiple diseases. Moreover, unlike existing PET-based techniques, 19F MRI allows for simultaneous imaging of multiple different fluorinated targets (*e.g.,* multispectral imaging). Challenges facing the modality include reduced spatio-temporal resolution, lower sensitivity compared to 1H MRI or PET, and the need for specialised equipment/expertise. Nevertheless, as technology improves, this form of molecular imaging will become indispensable in the precision medicine toolkit.
