# Supplementary material for: How to 19F MRI: applications, technique, and getting started
Source: BJR Open. 2023 Sep 29;5(1):20230019. doi: 10.1259/bjro.20230019 (PMC10636348; doi:10.1259/bjro.20230019)
Supplement: Supplementary file 1 — Supplementary S1. [file bjro.20230019.suppl-01.docx]

**Supplementary**

**S1.** Link to a general protocol for 19F MR image acquisition on a 7T Bruker system.

<https://www.protocols.io/blind/6A404CFDFEF611EDB9BC0A58A9FEAC02>
